# Supplementary material for: Single-site pyrrolic-nitrogen-doped sp2-hybridized carbon materials and their pseudocapacitance
Source: Nat Commun. 2020 Aug 4;11:3884. doi: 10.1038/s41467-020-17727-y (PMC7403304; doi:10.1038/s41467-020-17727-y)
Supplement: Supplementary file 1 — Supplementary Information [file 41467_2020_17727_MOESM1_ESM.pdf]

Supplementary Information

Single-site Pyrrolic-nitrogen-doped sp^2-hybridized Carbon Materials and Their Pseudocapacitance

Tian et al.

Supplementary Figures

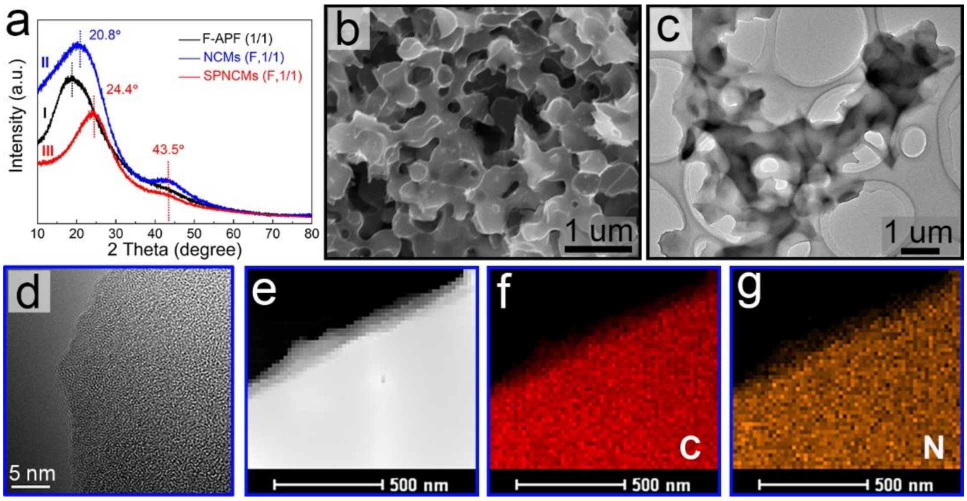

Supplementary Figure 1. Characterization of the samples. **a** XRD patterns of F-APF (1/1), NCMs (F,1/1) and SPNCMs (F,1/1). **b** SEM, **c** TEM, **d** HRTEM and **e** HAADF-STEM images and the elemental mapping images of **f** carbon and **g** nitrogen in SPNCMs (F,1/1). As shown in SEM (Supplementary Figure 1b) and TEM (Supplementary Figure 1c) images, microscopic structure of SPNCMs (F,1/1) exhibits mainly the feature of randomly assembled layers.

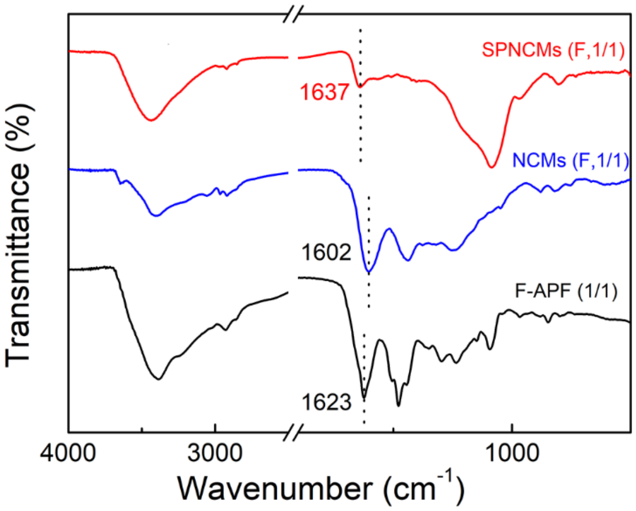

Supplementary Figure 2. FTIR spectra of F-APF (1/1), NCMs (F,1/1) and SPNCMs (F,1/1)

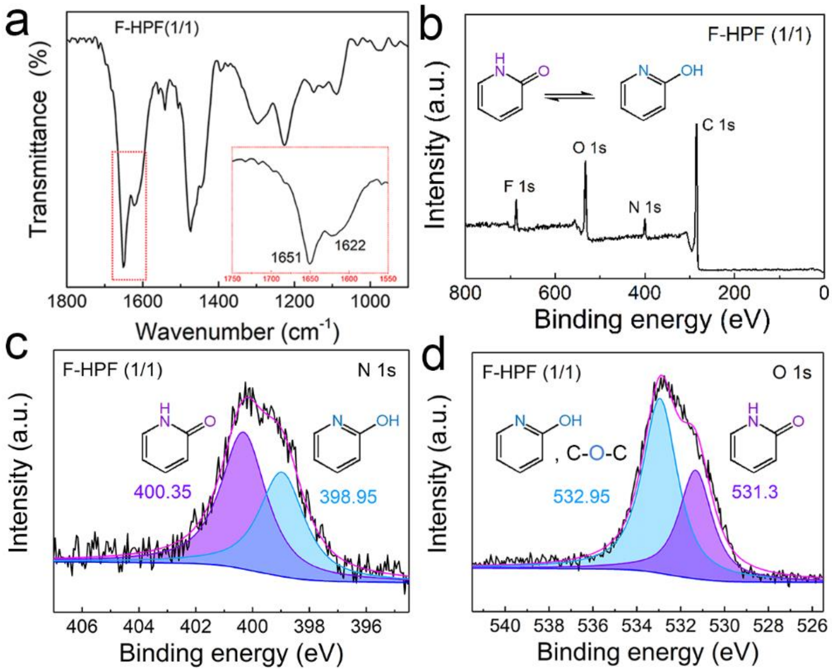

Supplementary Figure 3. The FTIR spectra and XPS spectra of F-HPF (F, 1/1). **a** The FTIR spectra (the inset shows the details of the red dashed box). **b** XPS survey spectrum (the inset is tautomeric schematic of α-pyridone and 2-hydroxypyridine configurations in the resin). **c, d** High-resolution XPS spectra for N 1s and O 1s of 3-fluorophenol-2-hyrdoxypyridine-formaldehyde resin, F-HPF (F, 1/1).

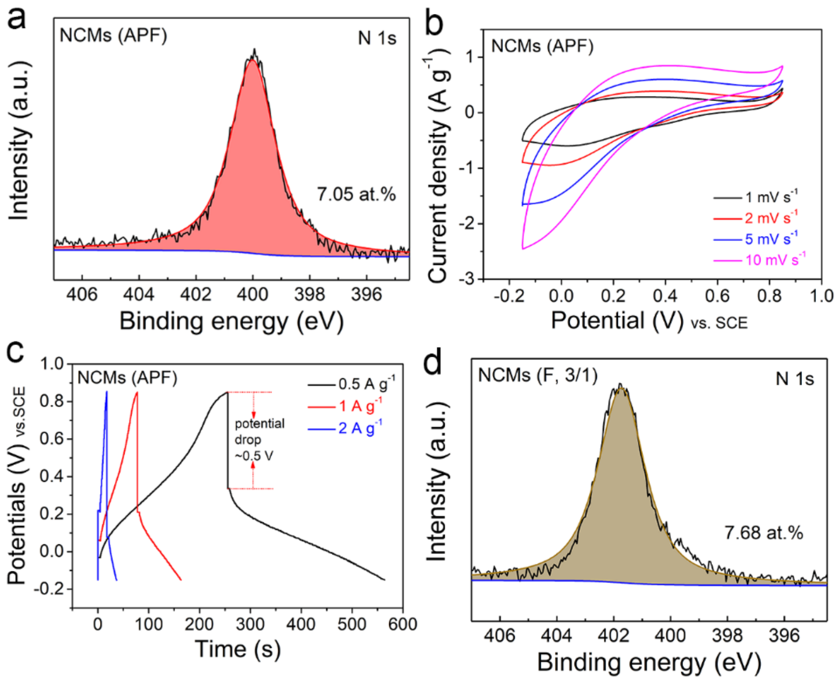

Supplementary Figure 4. The XPS spectra, CV and GCD curves of samples. **a** The high-resolution XPS spectra for N 1s of NCMs (APF) after activation. **b** CV tests at different scan rates from 1 to 10 mV s^{-1} and **c** GCD curves at different current densities from 0.5 to 2 A g^{-1} in 1.0 M H_2SO_4 solution of NCMs (APF). **d** The high-resolution XPS spectra for N 1s of NCMs (F, 3/1) after activation.

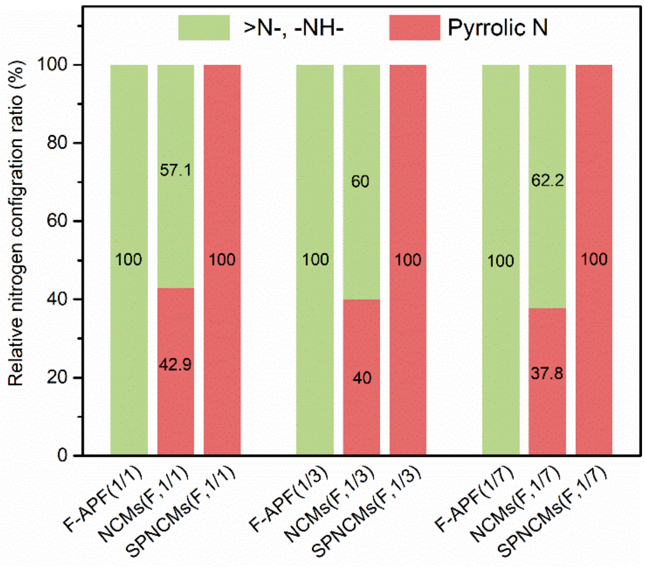

Supplementary Figure 5. The relative nitrogen configuration ratios in the samples.

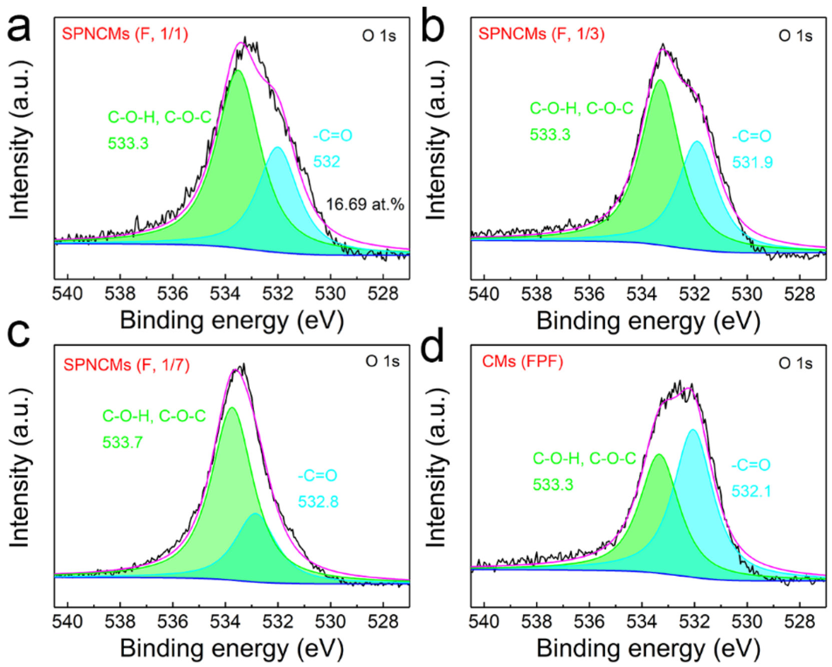

Supplementary Figure 6. High-resolution O 1s XPS spectra of the samples. High-resolution O 1s XPS spectra of **a** SPNCMs (F, 1/1), **b** SPNCMs (F, 1/3), **c** SPNCMs (F, 1/7) and **d** CMs (FPF).

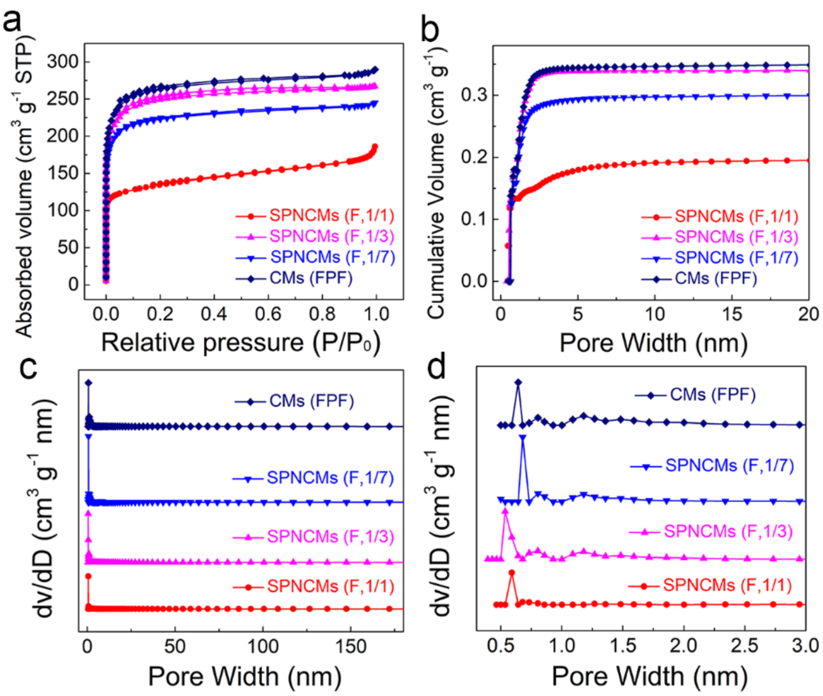

Supplementary Figure 7. N_2 adsorption/desorption analysis of the samples. **a** High-resolution, low-pressure N_2 (77.5 K) isotherms. **b** Cumulative pore volume and **c, d** pore-size distribution for N_2 adsorption. The cumulative pore volume in **b** and pore size distribution in **c, d** curves of these four carbon materials were plotted according to their corresponding nitrogen adsorption curves by an Original Density Functional Theory (ODFT) method.

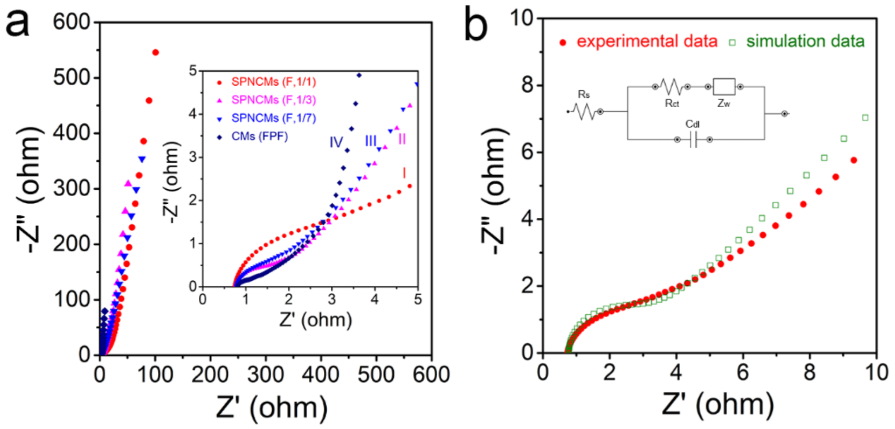

Supplementary Figure 8. Electrochemical impedance spectroscopy (EIS) for samples. **a** Complex-plane plots of electrochemical impedance spectroscopy (EIS) for SPNCMs (F, 1/1), SPNCMs (F, 1/3), SPNCMs (F, 1/7), and CMs (FPF) samples in frequency ranged from 0.01 to 100000 Hz in 1.0 M H_2SO_4. The inset shows the corresponding details at high-frequency ranges. **b** Nyquist plots (red dots) and Nyquist plot simulation (green boxes) of EIS for SPNCMs (F, 1/1); the inset displays the fitted equivalent circuit of the experimental data.

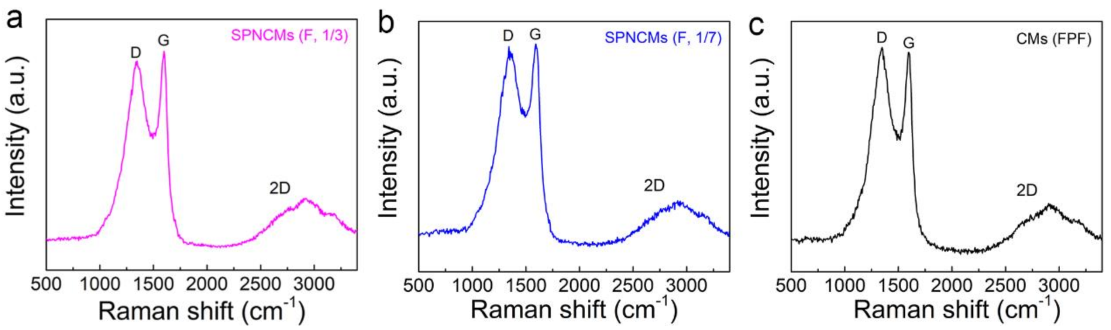

Supplementary Figure 9. Raman spectra of the samples. **a** SPNCMs (F, 1/3), **b** SPNCMs (F, 1/7) and **c** CMs (FPF).

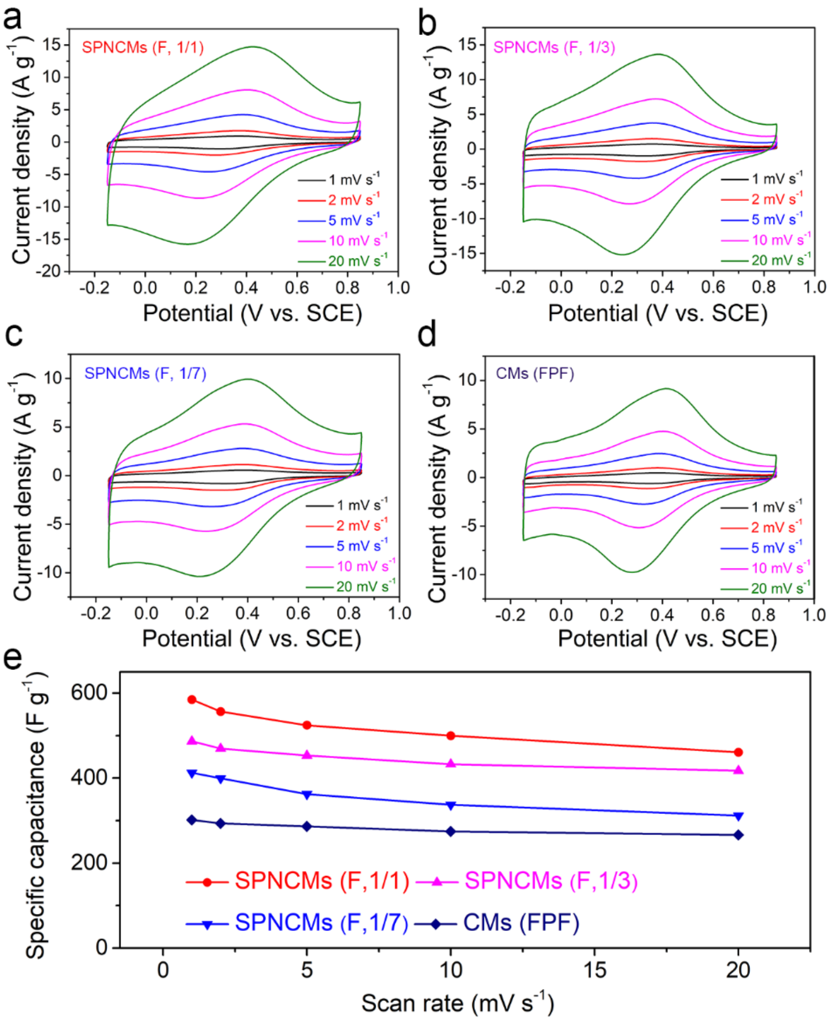

Supplementary Figure 10. CV tests of samples. **a-d** CV tests of SPNCMs (F, 1/1), SPNCMs (F, 1/3), SPNCMs (F, 1/7), and CMs (FPF) at different scan rates from 1 to 20 mV s^{-1} in 1.0 M H_2SO_4 solution in the potential window of -0.15 to 0.85 V vs. SCE. **e** The corresponding specific capacitance values of the above different carbon materials at different scan rates.

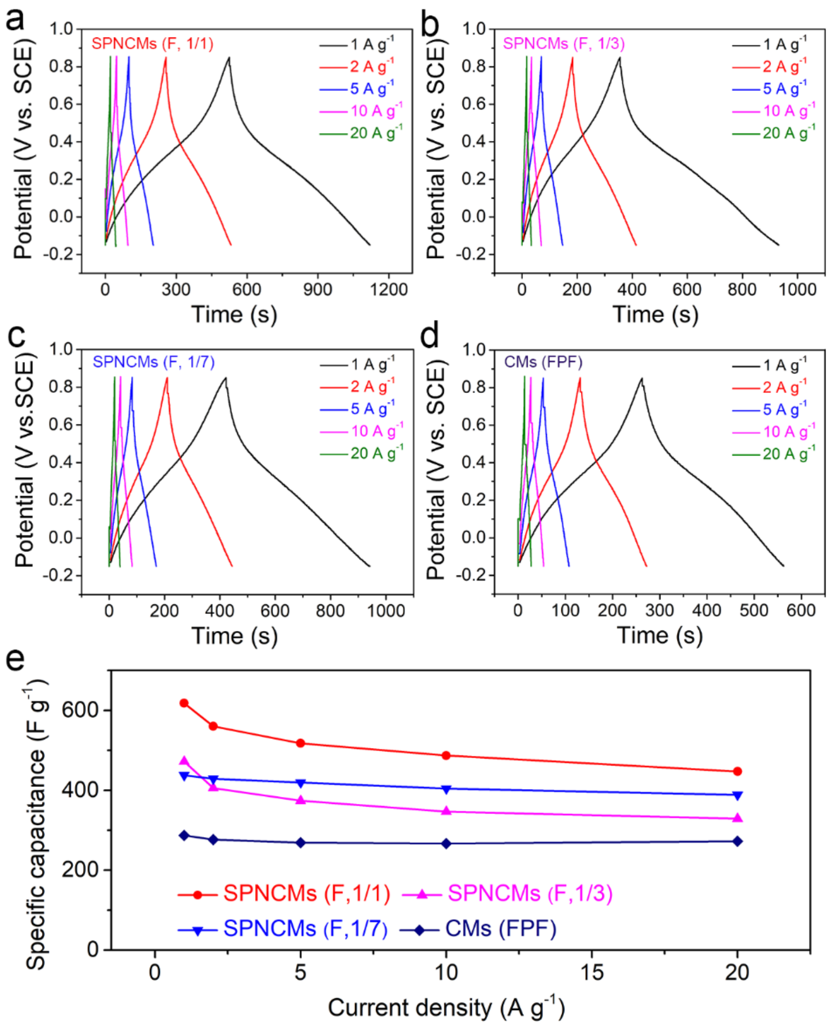

Supplementary Figure 11. GCD curves of samples. GCD curves of **a** SPNCMs (F, 1/1), **b** SPNCMs (F, 1/3), **c** SPNCMs (F, 1/7) and **d** CMs (FPF) at different current densities from 1 to 20 A g^{-1}. **e** The corresponding specific capacitance values of the above different carbon materials at different current densities.

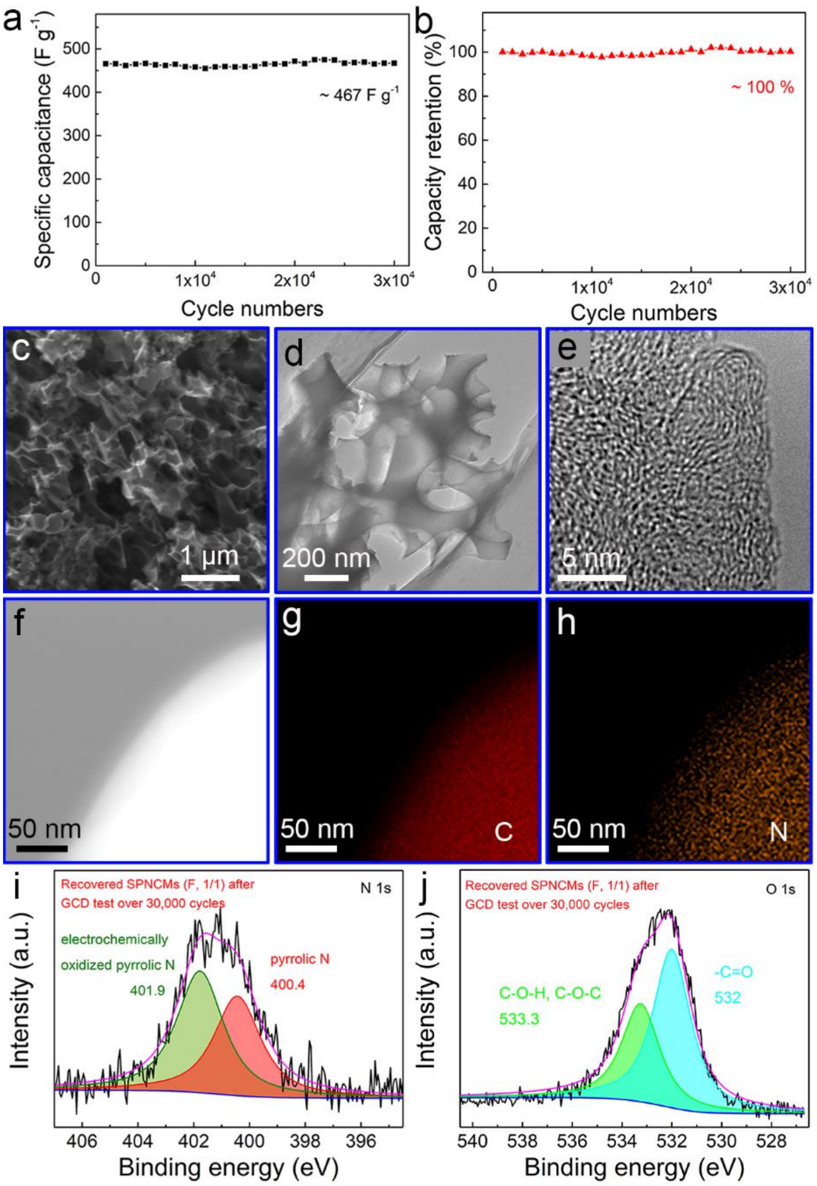

Supplementary Figure 12. Electrochemical stability of SPNCMs (F, 1/1). **a** Gravimetric capacitance and **b** capacity retention (%) of the SPNCMs (F, 1/1) electrodes in 1.0 M H_2SO_4 solution in a three-electrode system at a charge current of 10 A g^{-1} over 30,000 cycles. **c** SEM, **d** TEM, **e** HRTEM, **f** HAADF-STEM images, the elemental mapping images of **g** carbon and **h** nitrogen. High-resolution XPS spectra for **i** N 1s and **j** O 1s of the recovered SPNCMs (F, 1/1) electrodes after GCD test mentioned above over 30,000 cycles.

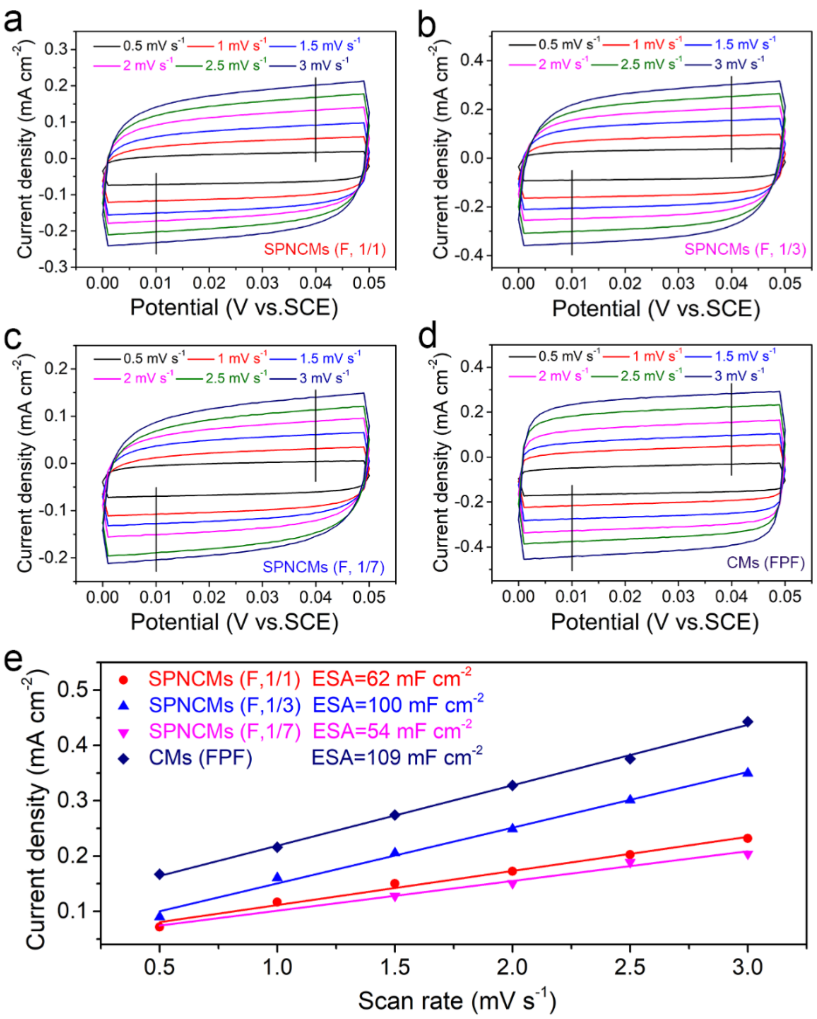

Supplementary Figure 13. EDLC of different carbon materials. Measurement of EDLC of different carbon materials by electrochemical surface area (ESA) without Faradic reaction in the voltage range (0-0.05 V vs. SCE). CV curves of **a** SPNCMs (F, 1/1), **b** SPNCMs (F, 1/3), **c** SPNCMs (F, 1/7) and **d** CMs (FPF) electrodes measured in 1 M H_2SO_4 at scan rates from 0.5 to 3.0 mV s^{-1}. **e** The corresponding ESA values determined by the linear curves of the discharge current density at 0.01 V (vs. SCE) vs. the scan rate. The capacitance of SPNCMs (F, 1/1) (0.493 mg cm^{-2}), SPNCMs (F, 1/3) (0.3825 mg cm^{-2}), SPNCMs (F, 1/7) (0.255 mg cm^{-2}) and CMs (FPF) (0.595 mg cm^{-2}) were measured to be 62, 100, 54 and 109 mF cm^{-2}, the corresponding EDLCs were about 125, 263, 211, 183 F g^{-1}, respectively.

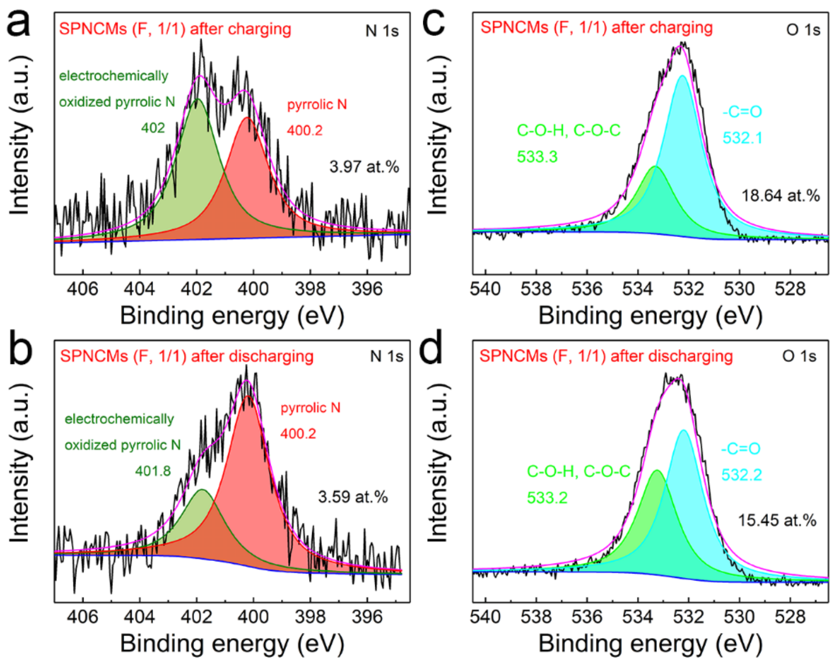

Supplementary Figure 14. High-resolution XPS spectra of samples. **a-d** High-resolution XPS spectra for N 1s (**a, b**) and O 1s (**c, d**) of the SPNCMs (F, 1/1) electrodes in 1.0 M H_2SO_4 solution in a three-electrode system at a charge current of 1.0 A g^{-1} after charging (**a, c**) and discharging (**b, d**).

Supplementary Tables

Supplementary Table 1. Binding energies and FWHM for N 1s in Figure 2 and Figure 3

Sample	NH-, ＞N- B.E.(eV)	-NH-, ＞N- FWHM (eV)	Pyrrolic N B.E.(eV)	Pyrrolic N FWHM (eV)	Figure
F-APF (1/1)	399.25	1.9	-	-	Figure 2c
NCMs (F, 1/1)	398.9	1.9	400.3	2	Figure 2d
SPNCMs (F, 1/1)	-	-	400.25	2	Figure 2e
F-APF (1/3)	399.53	1.9	-	-	Figure 3b
NCMs (F, 1/3)	398.97	1.9	400.57	2	Figure 3c
SPNCMs (F, 1/3)	-	-	400.25	2	Figure 3d
F-APF (1/7)	399.55	1.9	-	-	Figure 3f
NCMs (F, 1/7)	399	1.9	400.4	2	Figure 3g
SPNCMs (F, 1/7)	-	-	400.35	2	Figure 3h
Cl-APF	399.5	2	-	-	Figure 3j
NCMs (Cl)	398.9	1.9	400.3	2	Figure 3k
SPNCMs (Cl)	-	-	400.35	2	Figure 3l
Br-APF	399.5	1.9	-	-	Figure 3n
NCMs (Br)	398.9	1.9	400.5	2	Figure 3o
SPNCMs (Br)	-	-	400.4	2	Figure 3p

Supplementary Table 2. Binding energies and FWHM for N 1s and O 1s of 3-fluorophenol-2-hyrdoxypyridine-formaldehyde resin, F-HPF (F, 1/1) in Supplementary Figure 3

Sample	Pyridinic N B.E. (eV)	Pyridinic N FWHM (eV)	Pyridonic N B.E (eV)	Pyridonic N FWHM (eV)	-C=O B.E. (eV)	-C=O FWHM (eV)	-O-H/ C-O-C B.E (eV)	-O-H/ C-O-C FWHM (eV)
F-HPF (F, 1/1)	398.95	1.9	400.35	1.9	531.3	1.8	532.95	1.8

Supplementary Table 3. Binding energies and FWHM for N 1s of NCMs (APF) and NCMs (F, 3/1) in Supplementary Figure 4

Sample	B.E. (eV)	FWHM (eV)	B.E. (eV)	FWHM (eV)
NCMs (APF)	400	1.9	-	-
NCMs (F, 3/1)	-	-	401.7	2

Supplementary Table 4. Binding energies and FWHM for O 1s in Supplementary Figure 6

Sample	-C=O B.E.(eV)	-C=O FWHM .(eV)	-O-H/C-O-C B.E.(eV)	-O-H/C-O-C FWHM .(eV)
SPNCMs (F, 1/1)	532	1.85	533.5	1.9
SPNCMs (F, 1/3)	531.9	1.8	533.3	1.8
SPNCMs (F, 1/7)	532.8	1.85	533.7	1.9
CMs (FPF)	532.1	1.8	533.3	1.8

Supplementary Table 5. Binding energies and FWHM for N 1s and O 1s of SPNCMs (F, 1/1) in Supplementary Figure 12

Sample	Pyrrolic N B.E.(eV)	Pyrrolic N FWHM (eV)	Electrochemically oxidized pyrrolic N B.E.(eV)	Electrochemically oxidized pyrrolic N FWHM (eV)	-C=O B.E .(eV)	-C=O FWHM (eV)	-O-H/ C-O-C B.E.(eV)	-O-H/ C-O-C FWHM (eV)
After 30,000 cycles	400.4	2	401.8	2	532	1.8	533.25	1.8

Supplementary Table 6. C, N, O and F content of the samples determined by XPS analysis

Samples	C (at.%)	N (at.%)	O (at.%)	F (at.%)
F-APF (1/1)	75.98	4.27	13.59	6.17
NCMs (F, 1/1)	85.88	4.36	8.83	0.93
SPNCMs (F, 1/1)	78.4	4.22	16.69	0.7
SPNCMs (F, 1/3)	81.52	3.07	15.09	0.31
SPNCMs (F, 1/7)	76.17	1.51	22.32	0
CMs (FPF)	82.22	0	17.78	0

Supplementary Table 7. The feature, EDLC and pseudo-capacitance of the samples. EDLC and pseudo-capacitive contributions to the total specific capacity of SPNCMs (F, 1/1), SPNCMs (F, 1/3), SPNCMs (F, 1/7), and CMs (FPF) in three-electrode system at 1 mV s^{-1} in 1.0 M H_2SO_4 electrolyte

Samples	SSA (m^2 g^{-1})	Pyrrolic N (at.%)	Specific capacity (F g^{-1})	EDLC (F g^{-1})	Faradaic capacitance from oxygen species (F g^{-1})	Faradaic capacitance from pyrrolic nitrogen (F g^{-1})
SPNCMs (F, 1/1)	450	4.22	584.8	125	105.9	353.9
SPNCMs (F, 1/3)	950	3.07	486.7	263	100.8	122.9
SPNCMs (F, 1/7)	873	1.51	412.4	211	146.3	55.1
CMs (FPF)	880	0	301.5	183	118.5	0

Supplementary Table 8. Binding energies and FWHM for N 1s and O 1s of SPNCMs (F, 1/1) in Supplementary Figure 14

Sample	Pyrrolic N B.E.(eV)	Pyrrolic N FWHM. (eV)	Electrochemically oxidized pyrrolic N B.E.(eV)	Electrochemically oxidized pyrrolic N FWHM (eV)	-C=O B.E. (eV)	-C=O FWHM. (eV)	-O-H/ C-O-C B.E. (eV)	-O-H/ C-O-C FWHM (eV)
After charging	400.2	2	402	2	532.1	1.8	533.3	1.8
After discharging	400.2	2	401.8	2	532.2	1.8	533.2	1.85

Supplementary Table 9. Detailed synthesis parameters of the final nitrogen-doped carbon materials derived from 3-fluorophenol-3-aminophenol-formaldehyde (F-APF) co-condensed resin

Final nitrogen-doped carbon material after activation	The first-step pyrolyzed intermediates of co-condensated resin	Co-condensated resin	3-aminophenol / g	3-fluorophenol / g	HMT / g	H_2O / mL
SPNCMs (F,1/1)	NCMs (F,1/1)	F-APF (1/1)	0.1	0.1	0.1	80
SPNCMs (F,1/3)	NCMs (F,1/3)	F-APF (1/3)	0.05	0.15	0.1	80
SPNCMs (F,1/7)	NCMs (F,1/7)	F-APF (1/7)	0.025	0.175	0.1	80
CMs (FPF)	CMs (FPF) intermediate	FPF	0	0.2	0.1	80
NCMs (APF)	NCMs (APF) intermediate	APF	0.2	0	0.1	80
NCMs (F,3/1)	NCMs (F,3/1) intermediate	F-APF (3/1)	0.15	0.05	0.1	80

Supplementary Table 10. Detailed synthesis parameters of the final nitrogen-doped carbon materials derived from 3-chlorophenol-3-aminophenol-formaldehyde (Cl-APF) co-condensed resin

Final nitrogen-doped carbon material after activation	The first-step pyrolyzed intermediates of co-condensated resin	Co-condensated resin	3-aminophenol / g	3-chlorophenol / g	HMT / g	H_2O / mL
SPNCMs (Cl)	NCMs (Cl)	Cl-APF	0.1	0.1	0.1	80

Supplementary Table 11. Detailed synthesis parameters of the final nitrogen-doped carbon materials derived from 3-bromophenol-3-aminophenol-formaldehyde (Br-APF) co-condensed resin

Final nitrogen-doped carbon material after activation	The first-step pyrolyzed intermediates of co-condensated resin	Co-condensated resin	3-aminophenol / g	3-bromophenol / g	HMT / g	H_2O / mL
SPNCMs (Br)	NCMs (Br)	Br-APF	0.1	0.1	0.1	80

Supplementary Notes

Supplementary Note 1.

As revealed in Supplementary Figure 2, FTIR spectra provide some useful information about structural transformation from resin precursor to final SPNCMs. FTIR spectrum of F-APF (1/1) exhibits many characteristic bands including phenol O-H and N-H stretching vibration centered at 3387 cm^{-1}, aromatic C=C stretching at 1623 cm^{-1}, C-F stretching at 1296 cm^{-1}[1], -CH_2- asymmetric stretching at 2929 cm^{-1}, symmetric stretching at 2854 cm^{-1}, scissoring vibration at 1477 cm^{-1}, and C-O stretching at 1092 cm^{-1}, verifying successful synthesis of aminophenol-fluorophenol-formaldehyde resin. Compared with FTIR spectrum of F-APF (1/1), that of NCMs (1/1) shows weaker phenol O-H and N-H stretching vibration centered at 3400 cm^{-1}, and relatively weaker C-O stretching at 1092 cm^{-1}, demonstrating partial removal of oxygen species. In addition, both disappeared C-F stretching vibration at 1296 cm^{-1} and a shift of aromatic C=C stretching to a lower wavenumber (1602 cm^{-1}) verify an occurrence of thermally induced dehalogenation. Compared with FTIR spectrum of NCMs (1/1), that of SPNCMs (F,1/1) shows disappeared aromatic C=C stretching at 1602 cm^{-1} and two newly emerged peaks including C=O stretching vibration at 1637 cm^{-1} and C-O stretching vibration at 1086 cm^{-1}, demonstrating a transition of benzene into carbon structure and simultaneous oxidation of carbon materials to introduce possible phenol, carbonyl, and quinone groups in the KOH activation step.

Supplementary References

1. Moreira, M. A., et al. Theoretical and infrared studies on the conformations of monofluorophenols. *Journal of Molecular Structure* **1009**, 11-15 (2012).
